# β-Citronellol, an alcoholic monoterpene with inhibitory properties on the contractility of rat trachea

**DOI:** 10.1590/1414-431X20154800

**Published:** 2015-12-04

**Authors:** T.B. Vasconcelos, H.V. Ribeiro-Filho, L.T. Lucetti, P.J.C. Magalhães

**Affiliations:** Departamento de Fisiologia e Farmacologia, Universidade Federal do Ceará, Fortaleza, CE, Brasil

**Keywords:** Monoterpenes, Calcium channels, Smooth muscle, Airways

## Abstract

β-Citronellol is an alcoholic monoterpene found in essential oils such *Cymbopogon citratus* (a plant with antihypertensive properties). β-Citronellol can act against pathogenic microorganisms that affect airways and, in virtue of the popular use of β-citronellol-enriched essential oils in aromatherapy, we assessed its pharmacologic effects on the contractility of rat trachea. Contractions of isolated tracheal rings were recorded isometrically through a force transducer connected to a data-acquisition device. β-Citronellol relaxed sustained contractions induced by acetylcholine or high extracellular potassium, but half-maximal inhibitory concentrations (IC_50_) for K^+^-elicited stimuli were smaller than those for cholinergic contractions. It also inhibited contractions induced by electrical field stimulation or sodium orthovanadate with pharmacologic potency equivalent to that seen against acetylcholine-induced contractions. When contractions were evoked by selective recruitment of Ca^2+^ from the extracellular medium, β-citronellol preferentially inhibited contractions that involved voltage-operated (but not receptor-operated) pathways. β-Citronellol (but not verapamil) inhibited contractions induced by restoration of external Ca^2+^ levels after depleting internal Ca^2+^ stores with the concomitant presence of thapsigargin and recurrent challenge with acetylcholine. Treatment of tracheal rings with L-NAME, indomethacin or tetraethylammonium did not change the relaxing effects of β-citronellol. Inhibition of transient receptor potential vanilloid subtype 1 (TRPV1) or transient receptor potential ankyrin 1 (TRPA1) receptors with selective antagonists caused no change in the effects of β-citronellol. In conclusion, β-citronellol exerted inhibitory effects on rat tracheal rings, with predominant effects on contractions that recruit Ca^2+^ inflow towards the cytosol by voltage-gated pathways, whereas it appears less active against contractions elicited by receptor-operated Ca^2+^ channels.

## Introduction

The acyclic monoterpenoid β-citronellol (3,7-dimethyl-6-octen-1-ol; CAS number 106-22-9) has odor qualities that make it useful in the perfume industry (1). It is also used as a component of insect-repellent products. β-Citronellol has been used as a pesticide-active kairomone ingredient on food crops and ornamental plants to attract mites (2).

β-Citronellol has low toxicity with an oral median lethal dose of 3.45 g/kg for rats (3). β-Citronellol is naturally abundant as a volatile constituent responsible for the pleasant aroma and flavor of fruits such as *Vitis vinifera* L. (4). It is considered to be a Generally Recognized as Safe compound for food use. β-Citronellol belongs to a group of terpenoid-flavoring agents. The acceptable daily intake of β-citronellol is 0.5 mg/kg body weight with no toxicity at currently estimated levels of intake (5).

As an intermediary metabolic product, β-citronellol is found in the essential oil of *Cymbopogon citratus* (DC) Stapf. (Poaceae) and *Lippia alba* (Mill.) N.E. Brown. (Verbenaceae), aromatic plants that have antihypertensive properties (6,7). Hypotensive actions have been reported for β-citronellol, and vasodilation has been imputed to be part of its mode of action to decrease blood pressure in rats (8,9). Antagonism of transmembrane calcium ion (Ca^2+^) influx from the extracellular medium as well as inhibition of release of intracellular Ca^2+^ from Ca^2+^ stores appear to mediate its inhibitory effects on vascular smooth muscle (9). Inhibition of Ca^2+^ channels has been described for citral, farnesol, α-bisabolol and geraniol, compounds that are chemically related to β-citronellol (10-13).

Plants producing β-citronellol-enriched essential oils (e.g., lemon eucalyptus) are useful for the treatment of respiratory diseases, but knowledge regarding the mode of action is restricted to folk medicine. However, a prospective randomized double-blind controlled trial revealed that a spray application containing the essential oil of *Eucalyptus citriodora* Hook (Myrtaceae) improved upper respiratory symptoms in volunteers diagnosed with pharyngotonsillitis, viral laryngitis, or viral tracheitis (14). Mulyaningsih et al. (15) showed that β-citronellol is actively involved in the inhibitory effects of the essential oil of *E. citriodora* against multidrug-resistant bacterial pathogens. A more recent report showed that β-citronellol could be the active principle involved in the airborne inhibition of *Mycobacterium tuberculosis* (16). This finding raised the possibility of application of this essential oil through inhalation as therapy to impair recurrence of tuberculosis, which appears to be a recurrent public-health problem worldwide (16). Inhalation of infusions of *E. citriodora* is propagated widely in folk medicine as being effective against a wide range of respiratory complaints (17), but evidence to support its efficacy is lacking.

The present study was designed to determine the pharmacologic profile of β-citronellol on the contractility of isolated tracheal rings from rats. The emphasis was on the ability of β-citronellol to inhibit the contractile events mediated *via* recruitment of Ca^2+^ channels on smooth muscle cells (SMCs).

## Material and Methods

### Animals

Male Wistar rats (200-250 g) were obtained from populations maintained at the vivarium of the Departamento de Fisiologia e Farmacologia, Universidade Federal do Ceará (Fortaleza, CE, Brasil). Rats were maintained under conditions of constant temperature (22±2°C) with a 12-h light-dark cycle and free access to food and water. All animals were cared for in compliance with regulations set by the Brazilian National Council for Control of Experimentation with Animals. All procedures described herein were approved by the Animal Ethics Committee of the Universidade Federal do Ceará (protocol CEPA #28/12).

### Experimental setup for isolated trachea

Male rats were killed by cervical dislocation after anesthesia with tribromoethanol (250 mg/kg, *ip*). Tracheal rings were obtained by cutting (in a transverse direction) isolated trachea after careful dissection in a dish containing physiologic salt solution to remove adjacent tissues. From each trachea, three to four rings were prepared for maintenance in a 5-mL organ bath filled with physiologic salt solution at 37°C under continuous bubbling with 5% CO_2_ in O_2_ and pH 7.4. Each tracheal ring was suspended by two parallel stainless-steel rods passed through its lumen, as described previously (18). To stretch tracheal rings to a basal tension of 1 g, one stainless-steel rod was attached to a fixed pin in the organ bath and the other to a force transducer connected to a data-acquisition system (PowerLab 8/30, ADInstruments, Australia). Adjustments in basal tension were allowed during an equilibrium time of 1 h. Afterwards, contractions were induced by addition of 60 mM KCl directly to the organ bath. This procedure was repeated until two consistent reproducible contractions were elicited for each preparation. The magnitude of the final contraction served as a reference to express the subsequent contraction/relaxation responses induced for a given tracheal ring. In one set of experiments, electrical field stimulation (EFS) was employed to produce contraction of smooth muscle. In this case, tracheal preparations were disposed between stimulating electrodes suitable for organ-bath chambers (ADInstruments) and received electrical stimuli (LE 12406, Panlab, Spain) with pulse parameters of 50 V, 5 Hz, 5 ms, and 5 s.

### Concentration-response curves for β-citronellol on tracheal rings

Tracheal rings were challenged to contract in response to contractile stimuli (in general, a high potassium ion (K^+^) concentration (60 mM) or acetylcholine (ACh; 5 µM)). In the steady state of a given sustained contraction, concentration-effect curves were obtained by exposing preparations to increasing concentrations of β-citronellol, which was added cumulatively to the organ bath (12 min for each concentration). Control preparations received only the vehicle at an identical experimental time. In preparations contracted with KCl or ACh, concentration-effect curves to β-citronellol were constructed in the absence or presence of antagonists, as indicated below. In another set of experiments, a single concentration of β-citronellol was chosen and contractions were evoked to recruit a desired smooth muscle contractile pathway under certain circumstances. Other contractile agents were used and more experimental details for each protocol are provided in the Results and Discussion section.

### Solutions and drugs

The physiologic salt solution used was Krebs-Henseleit, which had the following composition: 118.0 mmol/L NaCl, 4.7 mmol/L KCl, 2.5 mmol/L CaCl_2_, 1.2 mmol/L MgSO_4_, 25.0 mmol/L NaHCO_3_, 1.2 mmol/L KH_2_PO_4_, 10.0 mmol/L glucose). Solutions with high KCl content involved addition of appropriate amounts of a 3-M KCl solution (in distilled water) directly to the organ bath to achieve the desired concentration. For some experiments, barium ions (Ba^2+^) substituted for Ca^2+^ in the physiologic salt solution.

(±)-β-Citronellol (95% purity; Code C83201), ACh (PubChem ID 24891113), atropine (ID 24890401), 5-hydroxytryptamine (ID 24278124), L-N^G^-nitroarginine methyl ester (L-NAME; ID 24278011), tetraethylammonium (TEA; ID 24277874), sodium orthovanadate (ID 24899708), capsazepine (ID 24277967), indomethacin (INDO; ID 24278173), A-967079 (CAS Number 1170613-55-4), HC-030031 (CAS Number 349085-38-7), thapsigargin (ID 24278762) and verapamil (ID 24277881) were purchased from Sigma-Aldrich (USA).

In general, stock solutions were prepared in distilled water and stored at -20°C. β-Citronellol was dissolved directly in physiologic solution containing 2% Tween 80 and sonicated immediately before addition in the bath chamber. The maximum concentration of the vehicle in the organ bath was 0.01% (*v/v*). Salts (all of analytical grade) were purchased from Sigma-Aldrich or Merck (Germany).

### Statistical analysis

Data are reported as means±SE. Half-maximal inhibitory concentration (IC_50_) and effective concentration (EC50) values were calculated by interpolation from semi-logarithmic plots, reported as geometric means (95% confidence interval), and compared using the Mann-Whitney U-test. Contractile responses of tracheal tissues were quantified and normalized as a percentage of the final contractile response to K^+^ (60 mM) obtained after the equilibration period or as a percentage of the ACh-induced sustained contraction as indicated. Significance of results was determined using ANOVA and, if significant, followed by a multiple comparison test. P<0.05 was considered significant.

## Results and Discussion

**Figure 1:**
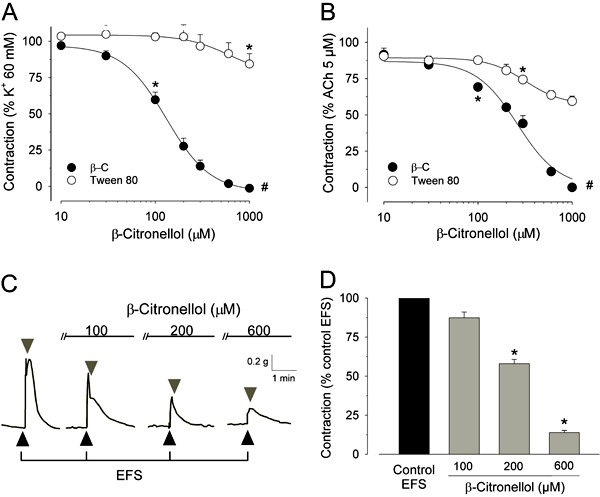
Myorelaxant and antispasmodic effects of β-citronellol on isolated rat trachea. *Panels A* and *B* show the inhibitory effects of β-citronellol (10-1000 µM β-C) added to the steady state of sustained contractions induced by K^+^ (60 mM; n=9; *A*) or acetylcholine (5 µM ACh; n=6; *B*). Vehicle alone (Tween 80; at the same concentrations employed to dissolve β-citronellol, i.e. 0.0002-0.01% *v/v*; open circles) induced relaxant effects that were significant at high concentrations, but small in magnitude in comparison with β-citronellol. Indicates the smallest concentration of β-citronellol or vehicle with a significant effect; P<0.05, Holm-Sidak test and # indicates a difference between treatments (vehicle *vs* β-citronellol) after two-way ANOVA. Typical traces and mean values for the inhibitory effects of β-citronellol (100-600 µM) on the transient contractions induced by electrical field stimulation (EFS; 50 V, 5 Hz, 5 ms, 5 s) are shown in *panels C* and *D*, respectively. A triangle indicates ON and an inverted triangle indicates OFF for the EFS. β-Citronellol was added 12 min before each contractile stimulus with EFS. *P<0.05 compared to control response induced by EFS in the absence of β-citronellol (ANOVA followed by the Holm-Sidak test; n=6).

In response to a high K^+^ concentration (60 mM; Figure 1A) or to ACh (5 µM; Figure 1B), rat tracheal rings developed sustained contractions that corresponded to 1142.4±187.6 (n=9) and 524.1±40.3 mg (n=6), respectively. If added at a steady-state contraction, β-citronellol (10-1000 µM) relaxed these sustained contractions fully with IC_50_ values to reverse K^+^-induced contraction [120.8 (89.1-163.8) µM; n=9] significantly lower than those needed to reverse contractions elicited by ACh [210.7 (175.9-252.3) µM; n=6; P<0.05, Mann-Whitney] (Table 1). Figure 1 also shows the slight inhibitory influence of the vehicle (Tween 80) in such contractions. Though significant at higher concentrations (especially for contractions induced by ACh), our findings argue against putative involvement of the vehicle in the relaxant effects induced by β-citronellol on rat tracheal rings.

**Table:**
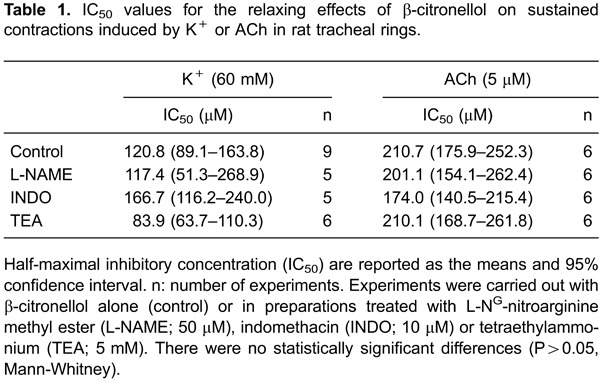

Smooth-muscle contraction was also evoked through EFS (Figure 1C,D). β-Citronellol (100-600 µM) inhibited the transient contractions induced by EFS with IC_50_ corresponding to 240.9 (207.8-279.3) µM (n=6). This value was higher than the IC_50_ needed to inhibit K^+^-induced contractions (P<0.05, Mann-Whitney), but not significantly different in comparison with the IC_50_ estimated for ACh-elicited contractions. Such results under EFS are consistent with the cholinergic nature of intramural neurons involved with the excitatory parasympathetic input towards tracheal smooth muscle (19).

One explanation for the higher pharmacologic potency of β-citronellol against contractions evoked by K^+^ could be related to the ubiquitous dependence of voltage-operated Ca^2+^ channels in the contractile effects induced by high K^+^ concentrations in SMCs, i.e. the "electromechanical coupling" (20). It has been shown that ACh enables transmembrane Ca^2+^ influx through L-type channels (especially in the SMCs of rat airways) but part of its action is secondary to the opening of chloride ion (Cl^-^) channels that can mediate membrane depolarization with further opening of Ca^2+^ channels gated by voltage (21). Nevertheless, the contractile response induced by cholinergic stimuli on tracheal smooth muscle occurs with substantial recruitment of other pathways (e.g., the metabotropic mechanisms related to receptor-operated Ca^2+^ channels) (21,22), which appear to be inhibited less by β-citronellol.

In this context, one set of experiments revealed that β-citronellol relaxed the contractions induced by sodium orthovanadate (0.3 mM) with IC_50_ of 243.0 (190.2-310.5) µM (n=6), which did not differ significantly from the IC_50_ required to relax ACh-induced contractions (P>0.05, Mann-Whitney). Sodium orthovanadate is a well-known tyrosine (Tyr) phosphatase inhibitor that indirectly shifts the kinase-phosphatase balance towards phosphorylation of Tyr kinases. Such findings are in accordance with the lower potency of β-citronellol against contractions evoked by metabotropic cascades if we consider that Tyr kinases have been reported to be downstream pathways in the contractile responses evoked by cholinergic agonists acting through G protein-coupled muscarinic receptors (23).

**Figure 2:**
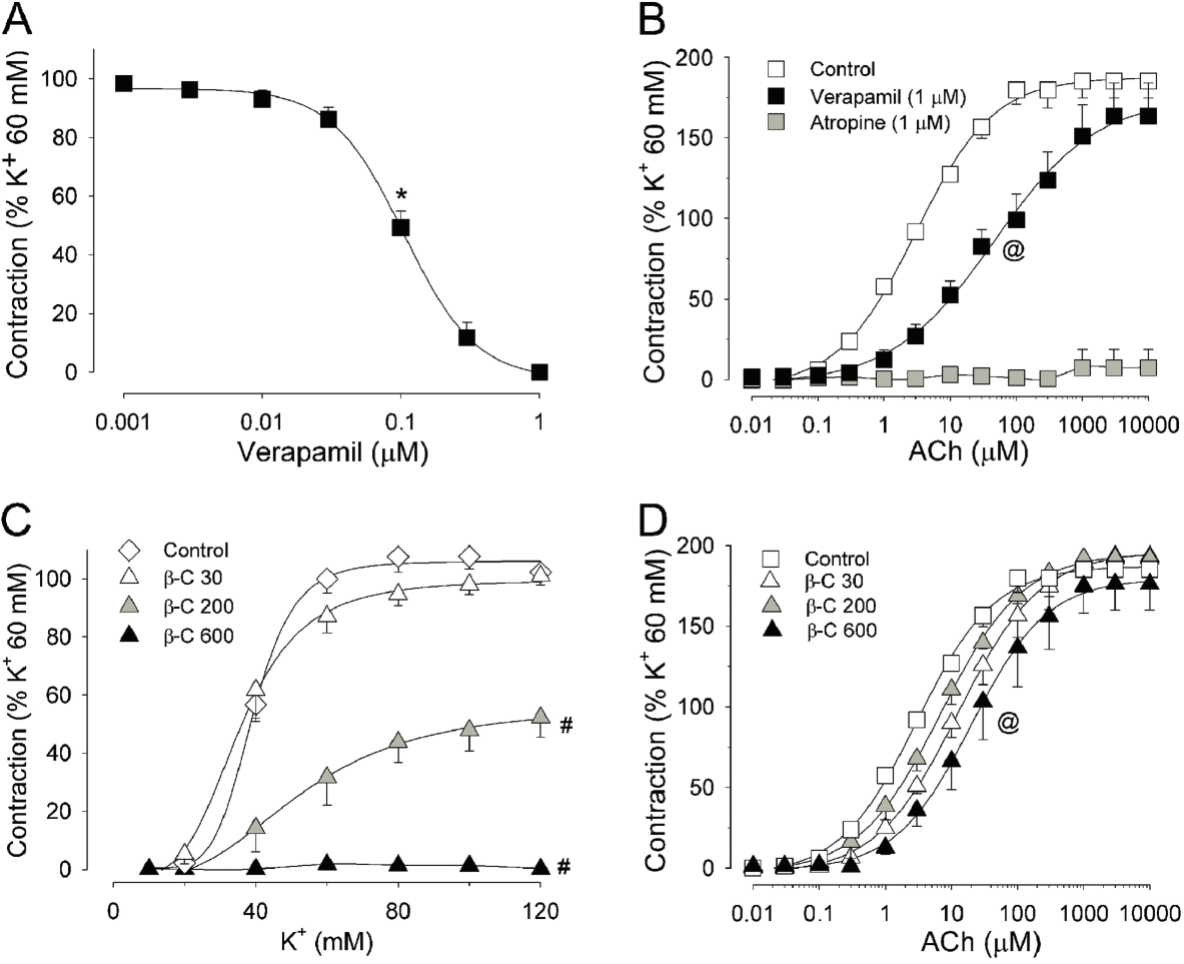
β-Citronellol preferentially inhibited the concentration-effect curves induced by high K^+^ concentrations in isolated rat trachea. *Panel A* shows the concentration-effect curve that determined 1 µM to be the verapamil concentration that could fully relax a sustained contraction induced by 60 mM K^+^. *Indicates the smallest concentration of verapamil with a significant effect; P<0.05, ANOVA followed by the Holm-Sidak test. In *panel B*, 1 µM verapamil only shifted to the right (indicated by @) the concentration-effect curve in response to increasing concentrations of acetylcholine (0.01 µM-10 mM ACh). The maximal effect induced by ACh was decreased significantly neither by verapamil (*B*) nor by β-citronellol (30-600 µM β-C; *panel D*), which produced only a similar rightward displacement of the concentration-effect curve of ACh (indicated by @). The positive control atropine fully inhibited the cholinergic response. *Panel C* shows the inhibitory effect of β-citronellol (30-600 µM) in the concentration-effect curve induced by increasing concentrations of K^+^ (10-120 mM). ^#^P<0.05 compared to control for the maximal effect, two-way ANOVA and Holm-Sidak test).

To test the hypothesis that β-citronellol has preferential inhibitory properties over contractions elicited by voltage-gated pathways, tracheal rings were subjected to treatment with verapamil (a phenylalkylamine compound possessing blockade properties on L-type Ca^2+^ channels in SMCs). First, a concentration-effect curve was constructed by adding verapamil to the steady-state contraction induced by 60 mM K^+^. Figure 2A reveals that at 1 µM verapamil fully relaxed the sustained contractions induced by K^+^, whereas Figure 2B shows that it only shifted to the right the concentration-effect curve induced by increasing concentrations of ACh (0.01 µM to 10 mM). Verapamil significantly augmented the EC_50_ of ACh from 1.9 (1.4-2.7) in control (n=8) to 17.8 (4.9-64.7) µM in the presence of verapamil (n=7; P<0.05, Mann-Whitney), but did not interfere significantly with the maximal effect achieved in the concentration-effect of ACh (Figure 2B).

Rightward displacement of the concentration-effect curve in response to ACh was also observed in tracheal rings maintained in increasing concentrations of β-citronellol (Figure 2D). At 600 µM β-citronellol, the EC_50_ in response to ACh was increased significantly to 46.5 (23.0-93.8) µM (n=6; P<0.05, Mann-Whitney). Just like verapamil, β-citronellol could not reduce the maximal contractile effect reached upon use of higher concentrations of ACh. The profile of the inhibitory action of β-citronellol against cholinergic contractions clearly differed from experiments in which increasing concentrations of K^+^ (10-120 mM) were used as contractile stimuli (Figure 2C). β-Citronellol produced a significant reduction in the K^+^-induced maximal effect at 200 µM, whereas complete inhibition was observed at 600 µM.

**Figure 3:**
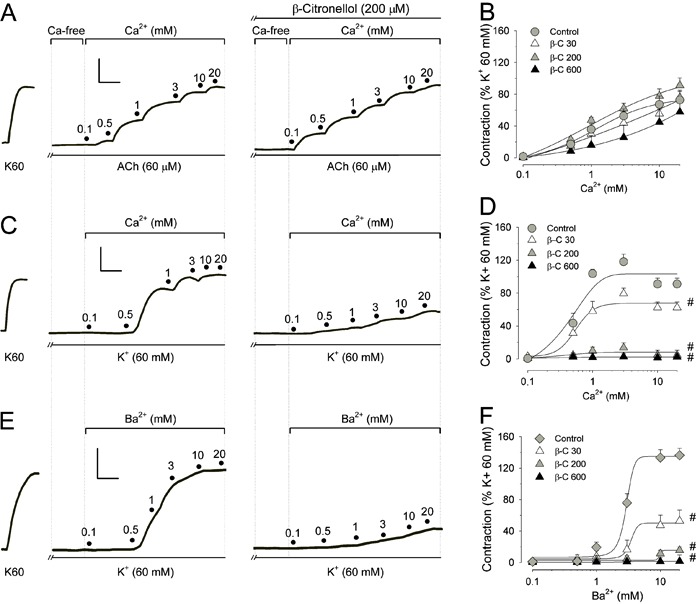
Inhibitory effects of β-citronellol on smooth-muscle contractions induced by recruitment of Ca^2+^ from the extracellular medium. Left panels show typical traces of experiments conducted in tracheal preparations maintained in Ca^2+^-free medium (containing 4 mM EGTA; in *A* with EGTA+10 µM verapamil) and stimulated with 60 µM acetylcholine (ACh) (*A*) or 60 mM K^+^ (*C* and *E*). Under such conditions, force developed by tracheal preparations remained at resting levels until addition of increasing concentrations of Ca^2+^ (0.1-20 mM; *A* and *C*). In tracheal preparations stimulated with K^+^, one set of experiments was conducted using Ba^2+^ (0.1-20 mM; *panel E*) instead of Ca^2+^. All these procedures were repeated in the presence of β-citronellol (30, 200 or 600 µM). Mean values are reported in the graphs of *panels B, D* and *F*. ^#^P<0.05 compared to control for the maximal effect using two-way ANOVA and the Holm-Sidak test. Calibrations: vertical, 0.3 g; horizontal, 3 min.

The preferential inhibitory profile of β-citronellol against contractions elicited by voltage-gated pathways was confirmed through additional experiments with tracheal preparations maintained in Ca^2+^-free medium containing ethylene glycol tetraacetic acid (EGTA; 4 mM). Under Ca^2+^-free conditions, addition of 60 mM K^+^ did not produce sustained contraction, and the contractile tonus of tracheal preparations remained at levels recorded under resting conditions. Still in the presence of high K^+^, cumulative addition of Ca^2+^ (0.1-20 mM; Figure 3C) promoted a gradual increase in contractile force and followed a concentration-dependent relationship (P<0.001, ANOVA). This response could be attributed to the depolarizing effects of K^+^ (which recruits Ca^2+^ from the extracellular *milieu* through voltage-gated Ca^2+^ channels) (18) and was decreased significantly by β-citronellol until complete blockade in the concentration range of 30 to 600 µM (n=6; Figure 3D). When Ba^2+^ (0.1-20 mM) substituted for Ca^2+^ in such procedures (Figure 3E), similar behavior was observed and tracheal preparations contracted in a β-citronellol-preventable manner (Figure 3F). It has been reported that Ba^2+^ can permeate through L-type Ca^2+^ channels and that it can substitute for Ca^2+^ in interactions with proteins of the contractile apparatus in SMCs (24-26).

Figure 3A shows the experimental setup in which tracheal preparations were stimulated with a high concentration of ACh (60 µM) under Ca^2+^-free conditions (medium containing EGTA and 10 µM verapamil). This pharmacologic approach aimed to diminish the influence of Ca^2+^ influx through voltage-operated channels when Ca^2+^ (0.1-20 mM) was added cumulatively to the extracellular medium. In contrast, the development of contractile force was seen probably because SMCs can also enable Ca^2+^ influx through metabotropic pathways such as the receptor-operated channels activated by phospholipase C-linked G proteins in response to ACh occupancy in muscarinic receptors (27). Under such conditions, β-citronellol was almost inert because the contraction in response to Ca^2+^ addition reached a magnitude comparable to that seen with β-citronellol-untreated control preparations (Figure 3B). Such findings reinforce the hypothesis that metabotropic mechanisms of contractions are inhibited to a lesser extent by β-citronellol.

Transmembrane influx of Ca^2+^ through store-operated Ca^2+^ channels ("capacitative Ca^2+^ entry") (28,29) can be also triggered under experimental conditions in preparations of rat trachea. This phenomenon can be activated in preparations maintained in Ca^2+^-free medium after treatment with thapsigargin (1 µM), a non-competitive inhibitor of sarcoplasmic/endoplasmic reticulum Ca^2+^ ATPase (30). Recurrent contractile stimuli in Ca^2+^-free conditions can be applied *via* activation of M_3_/M_2_ receptors by ACh (60 µM) and its downstream signal-transduction molecule inositol 1,4,5-trisphosphate, which elicits Ca^2+^ mobilization from the sarcoplasmic reticulum to the cytosol (31). Ca^2+^ reuptake is impaired by thapsigargin, so ACh-elicited emptying of internal stores of Ca^2+^ in the sarcoplasmic reticulum (60 µM) promotes activation of capacitative Ca^2+^ inflow (though the exact sequence of cell events in this pathway is not known). Irrespective of the amount of Ca^2+^ restored in the extracellular medium, SMCs can produce force even after removal of the cholinergic agent from the extracellular solution, a condition that resembles constitutive activity in pharmacologic receptors (32).

**Figure 4:**
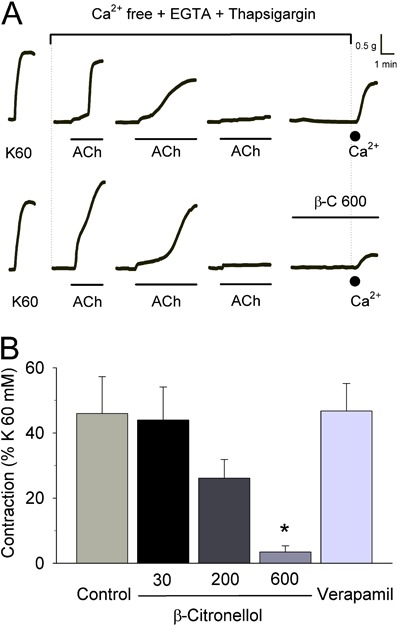
Inhibitory effects of β-citronellol on smooth-muscle contraction induced by store-operated capacitative Ca^2+^ entry. *Panel A*, typical traces of experiments conducted in tracheal preparations maintained in the presence of thapsigargin (1 µM) to produce activation of capacitative Ca^2+^ entry in response to repeated stimulation of tracheal rings with acetylcholine (60 µM ACh) in Ca^2+^-free medium (containing 4 mM EGTA). Emptying of intracellular Ca^2+^ stores was considered complete if a given stimulus applied with ACh did not produce a contraction. At this moment, ACh was removed from the extracellular medium by consecutive washings (4-5 times) with Ca^2+^-free solution. Afterwards, 2.5 mM Ca^2+^ (black circle) was added to the organ-bath solution, a procedure that elicited a contraction in control preparations (upper traces). In the presence of β-citronellol (30-600 µM; *panel B*), the contraction induced by Ca^2+^ restoration to the extracellular medium was decreased gradually and achieved a significant reduction at 600 µM β-citronellol (lower traces). *Panel B* is a graph with the means±SE values for these findings. Verapamil (1 µM) did not change the contraction induced by Ca^2+^ restoration. ;P<0.05 compared to control contraction in the absence of β-citronellol (Dunn's test).

In this context, a set of experiments was conducted to evaluate the inhibitory ability of β-citronellol against contractions induced by store-operated pathways (Figure 4A). Once emptying of intracellular Ca^2+^ stores could be confirmed by observation of unmeasurable responses, ACh was removed from the Ca^2+^-free extracellular *milieu* and 2.5 mM Ca^2+^ was added. Figure 4B shows that β-citronellol (30-600 µM; n=7) significantly reduced the magnitude of the contraction induced by addition of 2.5 mM Ca^2+^. Interestingly, verapamil (1 µM) did not change smooth-muscle contraction under these circumstances, a feature already reported for D-600 (an analog of this L-type Ca^2+^-channel blocker in bovine airway SMCs) (33). Thus, our findings show that β-citronellol can also inhibit smooth-muscle contractions evoked by capacitative Ca^2+^ entry. Considering that verapamil could not inhibit these contractile responses, it is unlikely that β-citronellol inhibited the contractions elicited by voltage-gated pathways in the same manner as verapamil (i.e. by direct blockade of L-type Ca^2+^ channels) (34).

**Figure 5:**
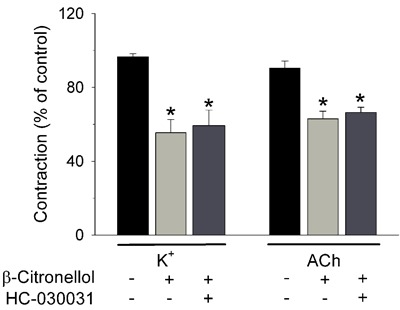
Evaluation of the involvement of the transient receptor potential ankyrin 1 (TRPA1) receptor in the myorelaxant effect of β-citronellol on rat trachea. Tracheal preparations were stimulated to contract in response to 60 mM K^+^ or 5 µM acetylcholine (ACh) and, at the steady state of the contraction, 100 µM β-citronellol was added in the absence or presence of the TRPA1 antagonist HC-030031 (20 µM). The tracheal contraction induced by K^+^ or ACh was reduced significantly by β-citronellol, an effect not influenced by the presence of the TRPA1 antagonist. Data are reported as means±SE. *P<0.05 compared to control contractions induced by K^+^ or ACh in the absence of both β-citronellol and HC-030031 (one-way ANOVA and Holm-Sidak test).

A few studies have reported that β-citronellol could be an agonist of human transient receptor potential vanilloid subtype 1 (TRPV1), a non-selective cation channel activated by capsaicin (35). Indeed, proteins of the TRP family have been imputed to be native store-operated Ca^2+^ channels in SMCs (36). However, this hypothesis cannot explain the actions of β-citronellol in rat trachea because the TRPV1 antagonist capsazepine (30 µM; n=9) did not change the relaxant effect induced by β-citronellol. In the presence of capsazepine, β-citronellol (200 µM) relaxed ACh-induced contraction to 49.0±4.8% (n=9), a magnitude deprived of a significant difference in comparison with the values observed when capsazepine was absent (55.2±1.8%; n=6). In addition, the β-citronellol analog citronellal activates transient receptor potential ankyrin 1 (TRPA1) proteins directly to repel insects (37). However, the TRPA1 antagonist HC-030031 (20 µM) did not antagonize the myorelaxant effects induced by 100 µM β-citronellol in tracheal preparations contracted with 60 mM K^+^ or 5 µM ACh (Figure 5). Similar results were obtained with another TRPA1 antagonist, A-967079 (10 µM; data not shown). Such findings preclude a putative role of TRPA1 as the mode by which β-citronellol induces relaxant actions in rat tracheal smooth muscle.

The inhibitory effects of β-citronellol were also tested in tracheal rings pretreated with L-NAME (50 µM), INDO (10 µM) or TEA (5 mM) but the IC_50_ values required to reverse K^+^- or ACh-induced contractions were not altered significantly (P>0.05, Mann-Whitney) (Table 1). The results with L-NAME and INDO suggest that β-citronellol did not recruit participation of the constitutive enzymes nitric oxide synthase or cyclooxygenase to produce its relaxant effects, respectively (38,39). In addition, putative opening of large-conductance Ca^2+^-activated K^+^ channels as the underlying mechanism to explain the actions of β-citronellol could also be discarded because TEA was inert against its relaxant effects (40).

In conclusion, the present study showed that β-citronellol has inhibitory properties on airway SMCs, a feature that should be considered in inhalatory therapies with β-citronellol-rich essential oils. β-Citronellol has higher potency to inhibit voltage-gated pathways. Our findings are in accordance with the notion that β-citronellol can antagonize transmembrane Ca^2+^ influx from the extracellular *milieu* to produce myorelaxant actions. β-Citronellol can also inhibit contractions mediated by metabotropic pathways, but with lower pharmacologic potency. It is unlikely that β-citronellol acts as a direct blocking agent on L-type Ca^2+^-channels on rat tracheal SMCs.
